# Links between Soil Fungal Diversity and Plant and Soil Properties on the Loess Plateau

**DOI:** 10.3389/fmicb.2017.02198

**Published:** 2017-11-07

**Authors:** Yang Yang, Yanxing Dou, Yimei Huang, Shaoshan An

**Affiliations:** ^1^College of Natural Resources and Environment, Northwest A&F University, Yangling, China; ^2^State Key Laboratory of Soil Erosion and Dryland Farming on the Loess Plateau, Northwest A&F University, Yangling, China

**Keywords:** soil fungal diversity, soil properties, plant properties, land use types, Loess Plateau

## Abstract

Previous studies have revealed inconsistent correlations between fungal diversity and plant/soil properties from local to global scales. Here, we investigated the internal relationships between soil fungal diversity and plant/soil properties on the Loess Plateau following vegetation restoration, using Illumina sequencing of the internal transcribed spacer 2 (ITS2) region for fungal identification. We found significant effects of land use types (Af, Artificial forest; Ns, Natural shrub; Ag, Artificial grassland; Ng, Natural grassland; Sc, slope cropland) on soil fungal communities composition, and the dominant phyla were *Ascomycota, Basidiomycota*, and *Zygomycota*, which transitioned from *Basidiomycota*-dominant to *Ascomycota*-dominant community due to vegetation restoration. The Chao1 richness, Shannon’s diversity and ACE indices were significantly influenced by land use types with the order of Ns > Af > Ng > Ag > Sc, and the total number of OTUs varied widely. In contrast, Good’s coverage and Simpson’s diversity indicated no significant difference among land use types (*p* > 0.05). Correlation analysis showed that plant and soil properties were closely related to fungal diversity regardless of land use types. In addition, soil organic carbon (SOC) and *H*_plant_ (plant richness, Shannon-Wiener index) were strong driving factors that explained fungal diversity. As revealed by the structural equation model (SEM) and generalized additive models (GAMs), fungal diversity was directly and indirectly affected by soil and plant properties, respectively, providing evidence for strong links between soil fungal diversity and plant and soil properties on the Loess Plateau.

## Introduction

Soil fungi play a crucial role in determining decomposition and nutrient cycling in terrestrial ecosystems (Bender et al., 2014; Rudgers et al., 2014; Tedersoo et al., 2014). Soil fungal community forms mutualistic symbiotic associations with plant and soil to improve absorption of nutrients (Voøíšková and Baldrian, 2013). Until recently, studies of fungal ecology (community composition and diversity) have been greatly limited by the problems of morphological identification (Montesinos-Navarro et al., 2016; Tedersoo et al., 2016). High throughput sequencing have provided a new perspective to study soil fungal ecology in ecosystems (Mueller et al., 2014; Horn et al., 2017).

Depending on the concept that “the environment selects and adaption” from Baas-Becking, the composition of soil fungal community are likely to be influenced by environmental variables (Aguilar-Trigueros et al., 2015; Filker et al., 2016). At a local scale, previous studies found that soil fungal diversity had an important influence on plant and soil properties (Rousk et al., 2010; Duchicela et al., 2013; Bender et al., 2014; van der Heijden et al., 2016). On one hand, higher fungal diversity and complex community composition enhance the decomposition rate of soil nutrients, which promote nutrients absorption and nutrient cycling (Kivlin et al., 2014; Hiscox et al., 2015; Yao et al., 2017a). On the other hand, plant provides a large amount of photosynthetic carbon for soil fungi growth, which affects soil fungal diversity via the obtained energy resources (Requena et al., 1997; Van Bruggen and Semenov, 2000; Vázquez et al., 2000; Sláviková et al., 2002; Ponge, 2013). For example, a larger number of studies reported that increasing plant richness resulted in the more richness of soil fungal diversity in rainforest ecosystems (Peay et al., 2013) and grassland (Brodie et al., 2003; Johnson et al., 2004). Besides, plant biomass (one plant property) has been reported to have positive (Van der Heijden et al., 1998), negative (Klironomos, 2002) or no (van der Heijden et al., 2016) effects on soil fungal diversity in different regions. Specifically, there have been strong interactions among soil fungal diversity, plant properties and soil properties in ecosystems, and little attention has been paid to soil fungal ecology in affecting plant and soil properties (Mueller et al., 2014; Urbanová et al., 2015; Sun et al., 2016; Ding et al., 2017). Thus, determining the links between soil fungal diversity and plant and soil properties could explore the microbiological mechanisms that how fungal diversity manipulated by plant and soil properties (Kivlin et al., 2014; García-Palacios et al., 2016; Schappe et al., 2017).

China’s Loess Plateau is one of the most deepest loess deposit and eroded area in the world (Fu et al., 2017). Last century, increasing population pressure and environmental damage issues resulted in ecological degradation in this region. To prevent the deterioration of the ecosystem, the government launched a series of ecological restoration engineering projects starting in 1980s (Deng et al., 2014; Feng et al., 2016). The “Grain for Green” vegetation restoration project or land use changes aim to rebuild the heavily damaged ecosystems. Now the Loess Plateau has become the most successful ecological restoration zone (Fu et al., 2017). Following the practice of vegetation restoration, croplands were converted into artificial forests (*Caragana korshinskii* and *Robinia pseudoacacia*) and grassland (artificial vegetation restoration), also converted into natural grassland and shrubs (natural vegetation restoration) by natural succession. In recent years, the responses of soil fungal diversity to vegetation restoration are only beginning to be explored, and responsiveness has been shown in some cases (Zeng et al., 2016; Zhang et al., 2016). Despite the fact that a large amount of literature have separately reported that fungal diversity, plant properties and soil properties changed following vegetation restoration (Feng et al., 2013; Deng et al., 2014; Zeng et al., 2016; Zhang et al., 2016), knowledge of the links between fungal diversity and plant and soil properties is still unclear in this region.

In this study, five land use types (artificial forest, Af; natural shrub, Ns; artificial grassland, Ag; natural grassland, Ng; and slop cropland, Sc) were selected on the Loess Plateau. The objectives are to test the links of plant and soil properties to fungal diversity regardless of land use type. There are three scientific questions: (i) how fungal diversity abundance, composition, and diversity altered by land use types; (ii) whether soil fungal diversity is linked to plant and soil properties, and (iii) if links do exist, what the relative contributions of plant and soil properties to soil fungal diversity are. To answer these questions, we postulated the following hypotheses: (1) soil fungal diversity and plant and soil properties affected by land use type; (2) plant and soil properties are associated with soil fungal diversity, and (3) plant and soil properties contribute to the fungal diversity on the Loess Plateau.

## Materials and Methods

### Sampling Areas

We carried out this study in a small watershed of the Yanhe catchment (36°23′-37°17′N, 108°45-110°28E) on the Loess Plateau located in the middle of the Yellow river. The study area occupies a total area of approximately 7,687 km^2^ with a semi-arid climate that has a heavy seasonal rainfall and periodic flooding. Hills cover 90% of the region (7,687 km^2^ in total area) with steep slopes (40%) by cliffs. Only 7% of this area is suitable for agriculture. The average annual rainfall from 1970 to 2000 was approximately 497 mm, and there are distinct rainy and dry seasons. The rainy season is from July to October, with the August rainfalls amounting for more than 20% of the annual total. The average annual temperature is from 5°C to 9°C along the elevation gradient. Most of the area lies at the altitude between 900 and 1500 m with loessal soil according to the Chinese Soil Taxonomy (Fu et al., 2000, 2011; An et al., 2013; Chen et al., 2013).

We selected 45 sites including five land use types: artificial forest, Af; artificial grassland, Ag; natural shrub, Ns; natural grassland, Ng; and slope cropland, Sc. These land use types initially developed from the similar parental material and the same climate but were changed by the different long-term land-use regimes. In addition, there were no signs of fire and natural disaster in this area during the past several decades according to historical sources. The loess is perfectly arable due to its fine grains, loose texture, and high content of mineral nutrients. In fact, it is the cradle of the ancient Chinese civilization with a long agricultural history (more than 6,000 years) in this area. The types of vegetation restoration in this area include artificial restoration (Af, Ag) due to the Grain for Green Project from 1999 and natural restoration (Ns, Ng) since 1938.

### Plant Sampling Design

**FIGURE 1:**
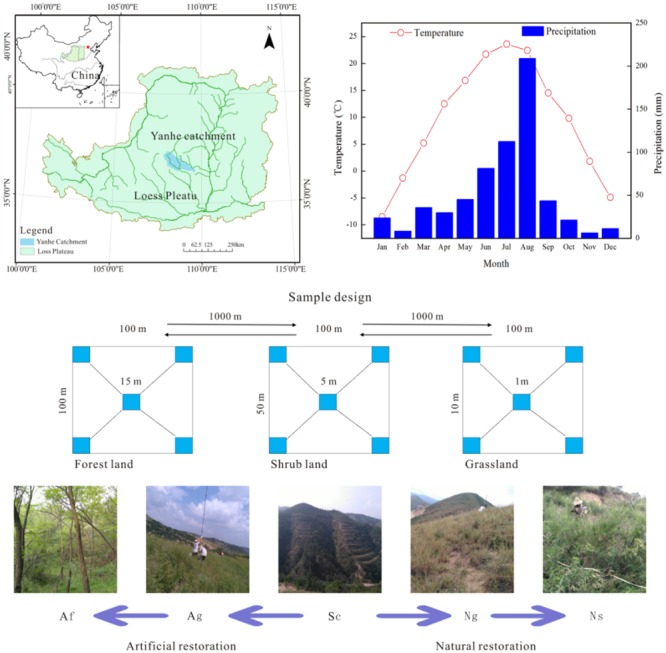
Location of the study area and layout of the plots among the different land use types. The pictures were generated by ArcMap Version 10.2 (http://www.esri.com/). These pictures were photographed by the authors in the Yanhe catchment during July 2016. Af, Artificial forest; Ns, Natural shrub; Ag, Artificial grassland; Ng, Natural grassland; Sc, Slop cropland.

This work was conducted during the peak of the growing season (September 2016). Sampling sites were located at least 1 km apart from each other. Each land use type had nine replicates, and we established a homogeneous 100 × 100 m plot (five replicate plots in the center of and around each site) (**Figure 1**). Samples of 15 × 15 m, 5 × 5 m, and 1 × 1 m were set in forest, shrub land, grassland and slope cropland. In the forest samples, we surveyed plant height (H) and all trees with >5 cm diameter at breast height (DBH) in each plot. We estimated the dry biomass by using the allometric model (Jackson et al., 2002). In addition, the dead trees and shrubs were also sampled initially, although they were not considered in the final calculations. Further, a total of 587 trees were measured across all of the plots. We established an allometric model equation with H and DBH. The allometric model to estimate the individual tree aboveground dry biomass (AGB) used the following equation:

(1) $$\begin{array}{c} \mathrm{AGB}\;=\;\exp(-0.{548+log}_{10}(\mathrm{BA})) \end{array}$$

where DBH refers to the diameter at breast height (cm), and BA refers to the basal area (cm^2^) calculated as π^∗^(DBH/2)^2^.

In shrub land, grassland, and slope cropland, we harvested all of the aboveground biomass. All of the litter was quantified after most of the aboveground parts had fallen. The litter was collected singly after all of the plant material accumulated on the ground surface within each plot. The aboveground biomass was oven-dried at 75°C for 48 h and then weighed.

In addition, the number of species, plant coverage, and individual plant H were investigated in each plot. The Patrick index (*S*_plant_) and Shannon-Wiener index (*H*_plant_) were calculated based on the plant community groups (Tilman et al., 1996; Keylock, 2005):

(2) $$\begin{array}{c} \mathrm{Patrick}\mathrm{index}(S_{\mathrm{plant}})\;=\;S \end{array}$$

(3) $$\begin{array}{c} Shannon-Wiener\mathrm{index}(H_{\mathrm{plant}})\;=\;-\sum P_{i}{\mathrm{LnP}}_{i} \end{array}$$

S represents the number of plant species, and plant species dominance (Pi) in each plot was calculated with the formula Pi = (relative abundance + relative height + relative plant cover)/3 (Tilman et al., 1996). The vegetation characteristics are summarized in Supplementary Table 1.

### Soil Sampling Design

Similarly, five replicates along an S-shape were sampled with a soil corer (10 cm in diameter), and after the visible roots, litter, and stones were removed, mixed five samples to obtain one composite one sample (0–20 cm). Each soil sample was divided into three parts: one part was immediately stored at -80 °C using liquid nitrogen for DNA analysis; one part was used to measure soil water content (SW, %) by oven drying in aluminum containers, and the last part was sieved through a 2-mm mesh, air-dried, and then analyzed for soil properties. Soil bulk density (BD, g⋅cm^-3^) was measured with a stainless-steel cylinder, and oven-dried at 105∘C for 48 h. In addition, a global positioning system (GPS) was used to obtain the basal gradient information, such as latitude, longitude, and altitude.

### Soil Property Analyses

Soil pH was measured in a 1:2.5 (v/v) soil:water:aqueous extract. Soil organic carbon (SOC, g kg^-1^) was measured by the K_2_Cr_2_O_7_-H_2_SO_4_ oxidation method (Nelson and Sommers, 1982). Soil total nitrogen (TN, g kg^-1^) was measured by the Kjeldahl procedure (UDK 140 Automatic Steam Distilling Unit, Italy) (Bremner and Mulvaney, 1982). Soil total phosphorus (TP, g⋅kg^-1^) was measured by the molybdenum antimony colorimetric method, and soil available phosphorus (AP, mg⋅kg^-1^) was extracted with 0.5 mol L^-1^ NaHCO_3_ and determined using the molybdenum-blue method (Olsen et al., 1982). Soil NH_4_^+^-N was measured using a Seal Auto Analyzer. Soil microbial biomass C and N (MBC and MBN, mg⋅kg^-1^) were measured by the fumigation-extraction method and calculated by using correction factors of 0.35 (kC) and 0.4 (kN), respectively (Brookes et al., 1985). Finally, each of the soil sample was performed in duplicate. The plant and soil properties among land use types are summarized in Supplementary Table 2.

### Soil Fungi Analyses

#### Soil DNA Extraction

First, DNA was extracted from 0.5 g freeze-dried soil samples using a MoBio Power Soil DNA Isolation Kit (MoBio Laboratories, Carlsbad, CA, United States) based on the manufacturer’s instructions. Second, we used a spectrophotometer (NanoDrop ND-1000, Wilmington, DE, United States) to purify the DNA in 260/280 nm and 260/230 nm absorbance ratios. Finally, we used a FLUOstar Optima (BMG Labtech, Jena, Germany) to concentrate the DNA, which was then stored at -80°C for further molecular analysis.

#### Quantitative PCR (qPCR) Analysis and Illumina MiSeq Sequencing

A quantitative PCR (qPCR) assay specific for the fungal internal transcribed spacer (ITS) region was used, as were ITS1F/ITS2F (ITS1F: 5′-GGAAGTAAAAGTCGTAACAAGG-3′, ITS2F: 5′-GCTGCGTTCTTCATCGATGC-3′) as primers (Fujita et al., 2001; Lau et al., 2007), which are considered to be the universal DNA barcode marker to identify fungal diversity (Blaalid et al., 2013). Quantification of the fungal ITS gene was performed using a PCR detection system (Applied Biosystems, Waltham, MA, United States), and the reaction mixture (20 μL) contained 2× FastFire qPCR PreMix (FastFire qPCR PreMix, Tiangen Biotech, China), 1× ROX Reference Dye, 1 μL of 1/10 diluted DNA, and 10 nM of each primer. The qPCR cycling conditions were 95°C for 5 min and 35 cycles of 95°C for 30 s, 50°C for 30 s, and then 75°C for 30 s, followed by the melting curve analysis. PCR amplicons were pooled in equimolar concentrations, and the primers and their dimers were separated by electrophoresis on a 1% agarose gel. The standard curve was generated using 10-fold serial dilutions of a plasmid containing the ITS gene insert. Illumina libraries were established using the MiSeq Reagent Kit v3 (Takara, China) based on the manufacturer’s instructions. Additionally, all of the soil samples were amplified in triplicate. The high-throughput sequencing data are available at the Shanghai Majorbio BioPharm Technology Company in the Sequence Read Achieve (SRA) database under accession number P20170119.

#### Processing of Sequencing Data

Raw sequences were built with various software tools^1^ (Obi et al., 2017). Paired-end reads were assigned according to the unique barcodes that were removed together with the primers and then calculated using QIIME software package^2^ (Quantitative Insights Into fungal Ecology, version 1.8.0). Subsequently, soil fungal diversity was analyzed with in-house Perl scripts. The abundance-based coverage estimator (ACE) and Chao1 estimator were calculated, and then, the rarefaction curves were plotted using MOTHUR.^3^ Phylogenetic diversity (PD) was estimated using Chao1 (Chao, 1984) and Faith’s indices (Faith, 1992; Turnbaugh et al., 2009). The weighted unifrac distance was measured with a unifrac metric. High quality sequences were clustered into operational taxonomic units (OTUs) based on 97% sequence similarity with UCLUST, which were analyzed with the National Center for Biotechnology Information (NCBI) BLAST against GenBank. Those with a minimum of 80% sequence similarity were preserved (Caporaso et al., 2010). Finally, a representative sequence from these OTUs was chosen on the 18S rRNA Silva reference database using the RDP classifier for phylogenetic information and taxonomic information (DeSantis et al., 2006)^4^.

### Statistical Analysis

Matrices of the pairwise taxonomic distance (Bray-Curtis) and the Euclidean distance among land use types were constructed using R package vegan (Version v.3.2.0).^5^ Based on the calculated Bray-Curtis distance, fitting of soil samples onto the NMDS graph and canonical correspondence analysis (CCA) were used to analyze the distribution of fungal community. Then, ANOSIM was performed using vegan in R (Ihrmark et al., 2012). The relationships between fungal diversity and plant and soil properties were evaluated by regression analyses performed at a 95% confidence interval (CI) in SPSS 21.0 for Windows (IBM Corporation, Armonk, NY, United States). The ternary sequence diagram of H_fungi_ (Shannon’s diversity index) and the Venn diagrams among land use types were plotted in Origin (Version 8.5).

To identify the contribution of plant and soil properties to fungal diversity, multivariate regression was applied (Ódor et al., 2006; Tedersoo et al., 2014). Additionally, generalized additive models (GAMs) were constructed with the ‘gam’ package in R. Prior to this analysis, soil properties (pH, SOC, TN, TP, AP, NH_4_^+^, MBC, and MBN) and plant properties (AGB, Coverage, *H*_plant_, and *S*_plant_) were treated as independent variables in the final model to explain the variation for fungal diversity. In addition, structural equation models (SEMs) were constructed in AMOS (Version 20.0), using Mantel R values as the input variation. Adequate model were determined by Fisher tests with a higher goodness-of-fit index (GFI) and lower Akaike information criteria (AIC) and root square mean errors of approximation (RSMEAs) (*p* < 0.05). Finally, based on theoretical knowledge, we attempted to construct a conceptual model to explore the links among soil fungal diversity and plant and soil properties.

## Results

### Fungal Community Structure and Diversity

In our findings, Shannon diversity index (*H*_plant_) and plant species richness (*S*_plant_) significantly affected by land use types. *H*_plant_ and *S*_plant_ in natural restoration (Ng, Ns) and artificial restoration (Af, Ag) were significantly higher than slope cropland (Sc) in terms of vegetation restoration (Supplementary Table 2).

**FIGURE 2:**
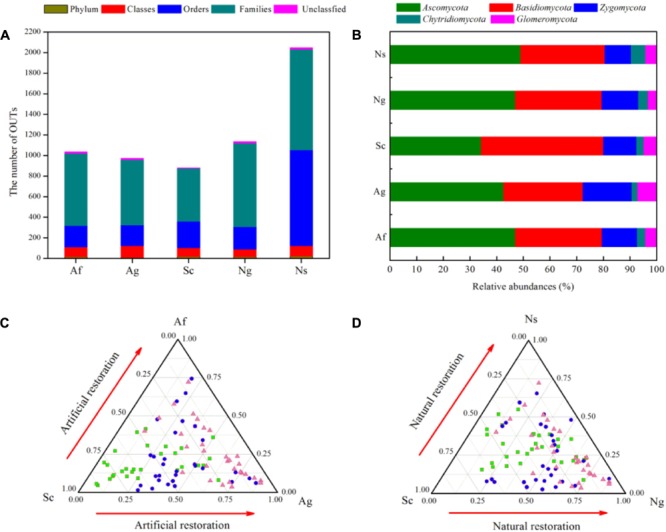
The number of OTUs and relative abundances (%) of fungal phyla among different land use types **(A,B)**. The ternary sequence diagram of *H*_fungi_ (Shannon’s diversity index) among the different land use types **(C,D)**. The gray circles represent *H*_fungi_ with no significant differences among the different land use types; the blue circles represent *H*_fungi_ with a significantly higher relative abundance, and the red circles represent *H*_fungi_ with a significantly lower relative abundance.

In total, 155,127 quality sequences were obtained from soil samples (Supplementary Table 3). Of all these sequences, 95.12% could be classified as fungal sequences from the NCBI database by BLAST hits, and 29,254-32,789 sequences were obtained per soil sample, belonging to 18 phyla, 111 classes, 223 orders, and 975 families. There were 582-927 phylotypes for all of the soil samples, with a mean of 767 phylotypes when grouped at the 97% similarity level. The dominant fungal phyla and their relative abundances were *Ascomycota* (36∼48%), *Basidiomycota* (31∼46%), and *Zygomycota* (10∼18%) among land use types. In addition, *Chytridiomycota* and *Glomeromycota* were minor phyla with lower relative abundances ranging from 2.13 to 5.27% and from 1.08 to 6.35%, respectively (**Figures 2A,B**). Although the relative abundance of fungal phyla fluctuated, the relative abundance of *Ascomycota* increased and the relative abundance of *Basidiomycota* decreased by land use types.

In addition, we found that the numbers of phylotypes and the calculated diversity, such as the Chao1 richness, Shannon’s diversity and ACE indices, were significantly affected by land use types (Supplementary Table 3). The Chao1, ACE and Shannon indices gradually increased in terms of vegetation restoration which showed natural restoration (Ng, Ns) and artificial restoration (Af, Ag) were significantly higher than Sc (*p* < 0.05), and the lower of Chao1, ACE and Shannon indices were observed in Sc with the order of Ns > Af > Ng > Ag > Sc, and the total number of OTUs varied widely. In contrast, Good’s coverage and Simpson’s diversity indicated no significant difference among land use types (*p* > 0.05). In addition, ternary plots showed that the distribution of the Shannon index differed among land use types (**Figures 2C,D**). Intriguingly, the Shannon index was significantly abundant in natural restoration (Ns, Ng) and artificial restoration (Af, Ag) compared to the Sc_._ Further taxonomical classification at the genus level revealed that more than 300 fungal genera were detected. Among them, 16 genera (with relative abundances greater than 1%) were detected in all of soil samples, and these genera in total accounted for more than 69% of the fungal sequences (Supplementary Table 4). Besides, *Mortierella, Fusarium* and *Guehomyces* were dominant genera, and their relative abundance varied from 15.16 to 20.43%, 6.05 to 14.74%, and 10.85 to 22.44%, respectively.

### Effect of Plant and Soil Properties on Fungal Diversity

**FIGURE 3:**
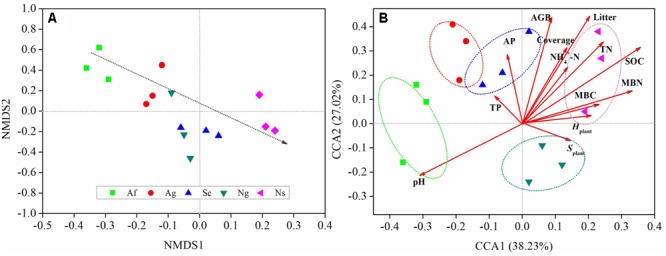
Non-metric multidimensional scaling plot (NMS) based on the calculated Bray-Curtis distance **(A)**. The distance between symbols is inversely proportional to the degree of similarity between the communities. Canonical correspondence analysis (CCA) of fungal diversity with plant and soil properties **(B)**. pH, soil pH value; SOC, soil organic carbon; TN,: soil total nitrogen; TP, soil total phosphorus; AP, soil available phosphorus; NH_4_^+^-N, soil NH_4_-N; MBC, soil microbial biomass C; MBN, soil microbial biomass N; AGB, aboveground biomass; *H*_plant_, Shannon-Wiener index; *S*_plant_, Patrick index.

Fungal community composition varied among land use types in NMDS plot based on the Bray-Curtis distance dissimilarity (**Figure 3A**). The NMDS plot showed that three randomly collected replicates from each land use type were usually closely located. The plot also indicated that the dominant of relative abundance of soil fungal community gradually decreased along the NMDS1 axis. Besides, soil fungal community among land use types were clearly separated from each other along the NMDS2 axis.

**FIGURE 4:**
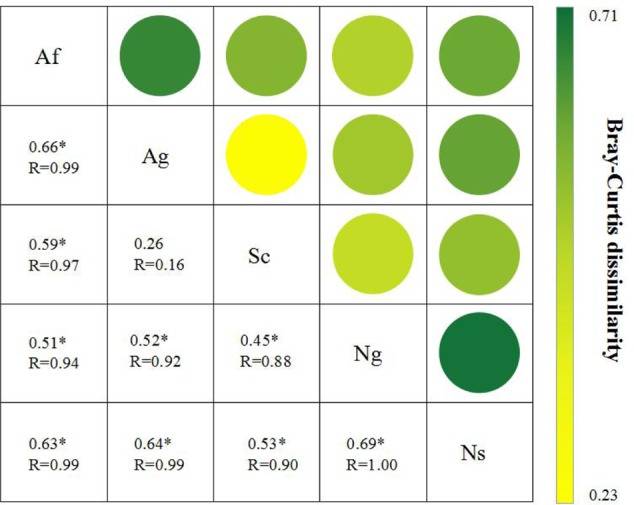
The Bray-Curtis dissimilarity of fungal diversity among different land use types. ^∗^Indicates that the fungal diversities were significantly different among land use types as detected by analysis of similarities (ANOSIM) using the Bray-Curtis distance. The *R*-values of the ANOSIM results, which were calculated based on 999 permutations, can be observed under the dissimilarity value.

Changes in soil fungal diversity were further depicted in the two-dimensional canonical correspondence analysis (CCA) plot by using Bray-Curtis distance (**Figure 3B**). Based on the result of the Mantel test and Spearman’s correlation coefficients (Supplementary Table 5), plant and soil properties were strongly related to fungal diversity and community structure by CCA. Fungal diversity affected by land use types along CCA1. The first two axes explained 65.25% of the total variation for fungal diversity, and the ordination of CCA showed significant differences among land use types. Among all of soil properties tested, soil pH and SOC were relatively near CCA1 axis, which explained 38.23% of the total variation, indicating that these two properties play an important role in shifting fungal diversity. In addition, MBC and MBN had an effect on fungal diversity along the CCA2 axis. Among all of plant properties tested, litter and AGB were relatively near the CCA1 axis. In addition, plant coverage had an effect on fungal diversity along the CCA2 axis. The analysis of similarities (ANOSIM) (**Figure 4**) showed that *R* value in artificial restoration (Af, Ag) and natural restoration (Ns, Ng) was significantly higher than Sc, suggesting that soil fungal diversity was significantly altered by land use types due to vegetation restoration.

### Structural Equation Model of Plant and Soil Properties for Fungal Diversity

**FIGURE 5:**
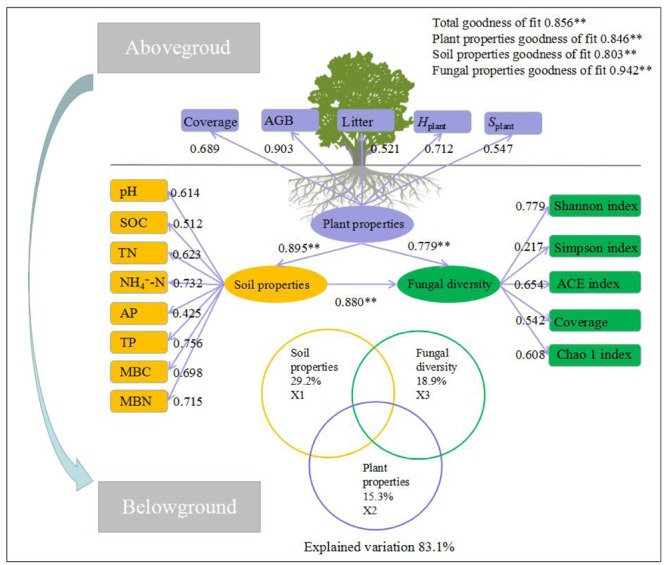
Structural equation model (SEM) among the plant and soil properties and fungal diversity. The standardized coefficient is given for the SEM. Values in rectangular frames denote the measurable properties. Values in ellipsoidal frames denote plant properties, soil properties and fungal diversity. The goodness of fit was greater than 0.8 for the SEM and the results of the generalized additive models (GAMs). The GAM analyses led to the following fractions: pure effect of soil properties (X1); pure effect of plant properties (X2); pure effect of fungal diversity (X3) (including the main phyla with relative abundance higher than 4%); and explained variation. The path coefficients and the explained variability were calculated after 999 bootstraps. Models with different structures were assessed using the goodness of fit statistic, a measure of the overall prediction performance.

Most of plant and soil properties are the key driving factors to explain the change of fungal diversity. Thus, variation partition analysis, stepwise regression, and GAMs were conducted to examine the relative contribution of plant and soil properties to fungal diversity (**Figure 5**). The combined stepwise regression and GAMs demonstrated that plant and soil properties were strongly related to fungal diversity. The variation of all the selected explanatory factors were 83.1%; soil properties variation were 29.3%, and plant properties variation were 16.7%. In addition, the variation in fungal diversity was primarily associated with plant and soil properties. Plant properties explained 16.7% of the variation, leaving 16.9% unexplained. Of all the selected soil properties, pH, SOC, TN, NH_4_^+^, AP, TP, MBC and MBN individually explained 61.4, 51.2, 62.3, 73.2, 42.5, 75.6, 69.8, and 71.5% of the variation observed, respectively.

Based on these analyses, the final SEM was fitted in order to describe the pathways of interaction among plant properties, soil properties, and fungal diversity (*F =* 35.26, df = 30, *p* < 0.01, GFI = 0.087, AIC = 116.35, RSMEA = 0.024). The final model explained 64.32% of the variation in fungal diversity (Supplementary Table 6). Further, fungal diversity was directly affected by soil properties (direct pathway effect was 0.651, and indirect pathway effect was 0.229, respectively) and directly affected by plant properties (direct pathway effect was 0.238, and indirect pathway effect was 0.539, respectively). In these direct and indirect pathway effects, SOC and *H*_plant_ had the higher direct or indirect pathway effects (0.283 and 0.280, respectively).

## Discussion

### Fungal Community Structure and Diversity

In this study, the Chao1 richness, Shannon’s diversity and ACE indices were significantly influenced by land use types with the order of Ns > Af > Ng > Ag > Sc, and the total number of OTUs varied widely. In contrast, Good’s coverage and Simpson’s diversity indicated no significant difference among land use types (*p* > 0.05). Thus, we can conclude that soil fungal diversity was strongly affected by land use types, supporting our first hypothesis. With *Leguminous* plants (*Robinia pseudoacacia, Caragana korshinskii*, and *Sophora viciifolia*) in terms of vegetation restoration, it (vegetation restoration) formed the dense root and consistently released amount of nutrients to improve the activity of soil fungi (Tiedje et al., 1999; Strickland and Rousk, 2010; Delgado-Baquerizo et al., 2013), resulting in the large changes of fungal diversity by land use types on the Loess Plateau.

Besides, large differences were observed in soil fungal community composition among land use types (**Figures 2A,B, 4**). The relative abundance of *Ascomycota* increased and the relative abundance of *Basidiomycota* decreased due to vegetation restoration, and the dominant phyla among land use types were *Ascomycota, Basidiomycota, Zygomycota*, which transitioned from *Basidiomycota*-dominant to *Ascomycota*-dominant community, indicating that fungal community transitioned from slow-growing oligotrophic fungi groups to fast-growing copiotrophic fungi groups. In addition, there was a higher Chao1, ACE and Shannon indices in artificial restoration (Af, Ag) and natural restoration (Ng, Ns) compared to slope cropland (Sc) (Supplementary Table 3). Similarly, plant and soil properties exhibited the same tendency with fungal diversity (Supplementary Table 2). These findings emphasize the importance of soil fungal diversity on plant and soil properties. Generally, soil fungal taxa occupied specific ecological niches according to available resources (Liggenstoffer et al., 2010; Barnard et al., 2013; Hartmann et al., 2015), and soil nutrients provided a suitable habitat for the activity of fungi (Landeras et al., 2005; Baldrian et al., 2012; Sun et al., 2015). In turn, soil fungal diversity greatly contributed to the process of nutrient decomposition (Vázquez et al., 2000; Schimel et al., 2007; Ponge, 2013; van der Heijden and Wagg, 2013). For example, higher fungal diversity and complex community composition enhanced the decomposition rate of soil nutrients (Kivlin et al., 2014; Hiscox et al., 2015; Yao et al., 2017b), which promoted to the nutrients absorption for plant (Dighton, 2006; Güsewell and Gessner, 2009; Dumbrell et al., 2010; Kembel et al., 2012; Bowles et al., 2014), leading to the same tendency of plant and soil properties with fungal diversity regardless of land use types.

### Plant and Soil Properties Affecting Fungal Diversity

In fact, our data clearly showed that plant and soil properties played an important role in soil fungal diversity, which supported our second hypothesis. In canonical correspondence analysis (CCA), fungal diversity was influenced by plant and soil properties regardless of land use types. This results do agree with previous studies in northeast China (Wang et al., 2015; Sun et al., 2016), while differ from the study conducted on the Loess Plateau (Zeng et al., 2016). Generally, soil fungal community on a large scale is determined by the latitudinal or latitudinal transect along with geographic distances, climatic conditions, mean annual precipitation (MAP), and mean annual temperature (MAT). However, our study conducted in unified climate and environmental conditions (even the uniform soil type), but with different land use types, which is credible for analyses of plant and soil properties affecting soil fungal diversity.

Among all the soil properties tested in affecting fungal diversity, soil pH and SOC play an important role in fungal diversity (**Figure 3**), which is in agreement with most previous studies (Wang et al., 2015; Sun et al., 2016; Zeng et al., 2016; Yao et al., 2017a,b). While there was no significant difference between fungal diversity and TP, AP (*p* > 0.05). Because P is primarily derived from the mechanical weathering of rock (Vitousek et al., 2010; Xiong et al., 2012), and thus we can conclude that there was little impact of soil fungal diversity on the P recycling in this region. Besides, NH_4_^+^-N was closely correlated with fungal diversity, while TN had no strong correlation with fungal diversity, which indicated that not all of N (TN and NH_4_^+^-N) contributed to fungal diversity. One route is that soil fungi enhance N availability by transforming N to more mobile forms, such as NH_4_^+^-N, which is easily dissipated by the activity of soil fungi, and in turn, these mobile N provide large energy to the activity of soil fungi, which contribute to increase soil fungal diversity (Dumbrell et al., 2010; Kembel et al., 2012; Maestre et al., 2013; Bowles et al., 2014).

Among all the plant properties tested, soil fungal diversity was significantly related to plant richness (Supplementary Table 5), supporting the findings from Peay et al. (2013), who found a significant correlation between plant richness and fungal diversity in tropical forests. Besides, Hooper et al. (2005) also found that there was a positive correlation between plant richness and fungal diversity. In this study, strong effect of plant richness on fungal diversity was found, and some feedback mechanisms are likely to be found at the plant species level (Veum et al., 2014; Hartmann et al., 2015; Riggs et al., 2015). One suggestion is that soil fungi form mutualistic symbioses with many plant species and are regarded as key organisms involved in nutrient cycling due to vegetation restoration. For instance, fast-growing plant species produce large amounts of litter and root exudates, which promote nutrient cycling and enhance fungal diversity. In contrast, slow-growing plant species produce lower amounts of litter and root exudates, which are typically related to nutrient cycling (Dumbrell et al., 2010; Kembel et al., 2012). Overall, this feedback mechanisms provide a form of mutualism between plant properties and fungal diversity.

In addition, plant and soil properties explained most variation of fungal diversity by GAMs and SEM (**Figure 5** and Supplementary Table 6), which supported our third hypothesis. There is a reasonable assumption that structural and compositional variations in plant and soil properties would be synchronous, but the following question remained: Do soil properties play a greater role than plant properties in fungal diversity? So SEM provide evidence that plant and soil properties were highly related to fungal diversity. No surprising, we found that soil properties were related to fungal diversity as direct effects, while plant properties had an indirect effect on fungal diversity. In these direct and indirect pathway effects, SOC and *H*_plant_ had higher direct or indirect pathway effects (0.283 and 0.280) on fungal diversity. Giving the reasonable explanation, fungal diversity was indirectly influenced by plant properties via the resource partitioning and the rates of nutrient supply (Pérez-de-Mora et al., 2006; Peiffer et al., 2013; Osakabe et al., 2014; Zechmeister-Boltenstern et al., 2015). In contrast, soil properties directly contributed to the decomposition of organic matter (Cullen, 2008; Rousk et al., 2010; Aguilar-Trigueros et al., 2015; Mueller et al., 2016), and then resulted in the increase of soil fungal diversity (Waring et al., 2013; Zhu et al., 2014; Zuppinger-Dingley et al., 2014). Although our results demonstrate that plant and soil properties were the main driving factors that explained soil fungal diversity, the influence of human disturbance and harvesting needs to be considered and investigated more thoroughly. Therefore, the improvement of plant and soil quality may provide some management measures for on soil fungal diversity the Loess Plateau.

### Conceptual Model of Plant-Soil-Fungal Progress

**FIGURE 6:**
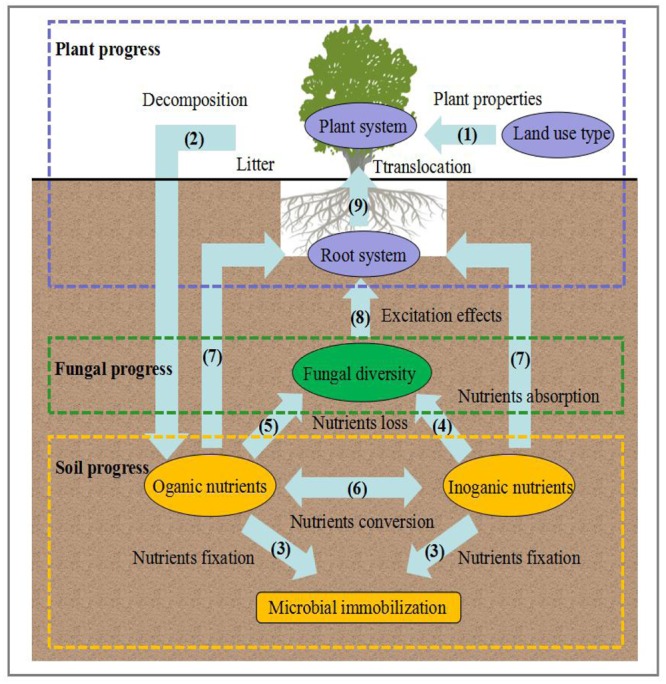
Conceptual model of the expected causal relationships among fungal diversity composition and soil and plant properties.

Our data emphasized the importance of plant and soil properties and their effects on fungal diversity on the Loess Plateau. Although we cannot rule out the mechanisms proposed in the field site, the underlying links between fungal diversity and plant and soil properties have been analyzed by the conceptual model (**Figure 6**). In this section, the possible explanations and the progress are discussed, resulting in three potential scenarios:

Plant progress: Land use type/changes alter plant properties. Subsequently, litter is always considered to be the main pathway of nutrient input from the turnover of fine roots and the surface soil layer, which is decomposed into organic and inorganic nutrients. This progress can clearly be explained by the changes in land use types, plant diversity, and plant productivity, which contribute to the increases of nutrients, and these increasing nutrients would provide natural resources for the growth of fungal community.

Fungal progress: Due to a mount of litter and plant productivity, the turnover rates of nutrients increased, resulting in the higher fungal diversity. In this progress, two main mechanisms can be introduced: one is the direct effects on plants, which form mutualistic relationship via root-associated organisms through fungal community, while the other is the indirect effects that occurred from the alteration of nutrient supply rates by the free-living fungal community.

Soil progress: A wide range of fungal community form intimate symbiotic associations with plant roots. Once established, the links between fungal diversity and plant and soil properties resulted in a common construction and relatively stable mycorrhizal network from the large abundance of fungi. In this network, fungal symbionts from leguminous plants contribute to N absorption (N-fixing progress) and even promote nutrient cycling in plant-soil-fungal system. Overall, the ecological processes suggest the strong interaction among soil fungal diversity, soil properties and plant properties on the Loess Plateau.

## Conclusion

In summary, we examined the links between fungal diversity and plant and soil properties on the Loess Plateau. Our findings revealed that land use types have a large influence on plant and soil properties and the relative abundances of dominant fungal groups. Plant and soil properties, such as SOC and *H*_plant_, were closely related to fungal diversity, which can be regard as the primary factors explaining soil fungal diversity regardless of land use types. This highlights the importance of plant and soil properties to soil fungal diversity. In addition, soil properties have a direct effect on fungal diversity, and plant properties have an indirect effect on fungal diversity according to the SEM and GAMs. Finally, the possible explanations and the progress regarding the links between fungal diversity and plant and soil properties were discussed in conceptual model. Thus the future research should focus more on the inner mechanisms such as the role of functional genes in the plant-soil-fungal system on the Loess Plateau.

## Author Contributions

YY, YD, YH, and SA conceived and designed this study. YY and YD performed the field trip, sample analysis and data analysis. YY drafted the original manuscript. SA provided very constructive suggestions for revisions. All the authors approved for publication.

## Conflict of Interest Statement

The authors declare that the research was conducted in the absence of any commercial or financial relationships that could be construed as a potential conflict of interest.

## Footnotes

1 https://en.wikipedia.org/wiki/Fastq

2 http://qiime.org/

3 http://www.mothur.org/

4 http://www.arb-silva.de

5 https://www.r-project.org/
