# Supplementary material for: Links between Soil Fungal Diversity and Plant and Soil Properties on the Loess Plateau
Source: Front Microbiol. 2017 Nov 7;8:2198. doi: 10.3389/fmicb.2017.02198 (PMC5682006; doi:10.3389/fmicb.2017.02198)
Supplement: Supplementary file 1 [file Table_1.DOC]

**Links between** **soil fungal diversity** **and plant and soil properties on the Loess Plateau**

*Yang Yang^1^, Yanxing Dou^2^, Shaoshan An^1,2*^*

*1* *College of Natural Resource and Environment,* *Northwest A&F University, Yangling 712100, China*

*2* *State Key Laboratory of Soil Erosion and Dryland Farming on the Loess Plateau, Northwest A&F University, Yangling 712100, China*

*Corresponding author at: College of Natural Resource and Environment, Northwest A&F University. Rd. Xinong No.26, Yangling, Shaanxi, 712100, China.Tel: +86-29-87012871; Fax: +86-29-87012210. *E-mail address*: [shan@ms.iswc.ac.cn](mailto:shan@ms.iswc.ac.cn) (S. S. An)

**Suppl. Table 1** Characterization of different land use types, including general vegetation structure. Values represent mean and standard deviation (in braquets) for each land use type. Values followed by the same letter in a given row are not significantly different at *p*<0.05 (*LSD* Fisher). The same below.

| Item | Artificial restoration | | Slope cropland  (Sc) | Natural restoration | |
| --- | --- | --- | --- | --- | --- |
|  | Artificial forest  (Af) | Artificial grassland (Ag) |  | Natural grassland (Ng) | Natural shrub  (Ns) |
| Land use regime | No significant logging during past decades with approximate 20 years age in terms of Grain-for-Green | Current heavy logging and cradled without cow and goat | Grazing blocked by fencing mainly in shallow ploughing to preserve heavy water loss and soil erosion | No significant logging or livestorage grazing in the past decades, respectively | Grazing blocked and no significant logging with fertile soil in the surface soil layer, Due to Natural Forest Protection Projects |
| Dominant plant species | *Robinia pseudoacacia* | *Medicago sativa* | *Artemisia giraldii, Phragmites australis* | *Stipa bungeana* | *Sophora viciifolia* |
| Litter thickness (mm) | 21.81±3.02^a^ | 2.02±0.47^c^ | 0.50±0.08^d^ | 15.53±3.15^b^ | 17.8±2.17^b^ |
| Tree cover (%) | 67.73±5.21 | - | - | - | - |
| Shrub cover (%) | 12.32±2.13^a^ | - | - | - | 56.97±6.32^b^ |
| Herbaceous cover (%) | 35.41±5.16^c^ | 32.10±2.89^c^ | 85.55±2.09^a^ | 86.93±6.74^a^ | 43.29±3.26^b^ |
| Maximum H (cm) | 1247.51±233.21^a^ | 39.21±8.02^d^ | 35.61±5.47^d^ | 105.42±13.25^c^ | 369.68±65.44^b^ |
| Maximum tree DBH (cm) | 11.33±3.02 | - | - | - | - |
| Species number* | 9.31±1.98^b^ | 6.86±0.85^c^ | 3.34±0.58^d^ | 16.75±2.17^a^ | 15.37±2.13^a^ |
| Herbaceous biomass (g·m^-2^) | 62.34±9.25^c^ | 34.50±5.69^d^ | 96.31±13.05^a^ | 113.57±10.14^a^ | 86.23±11.03^b^ |
| Shrub biomass (g·m^-2^) | 12.36±3.21^b^ | - | - | - | 1025.67±102.49^a^ |
| Tree biomass (g·m^-2^) | 1623.74±156.27 | - | - | - | - |
| Dominant value | 5.14±0.85^b^ | 2.01±0.45^c^ | 0.59±0.12^c^ | 7.89±1.37^ab^ | 9.23±1.20^a^ |

**Suppl. Table 2** Selected plant and soil properties among different land use types.

| Item | Artificial restoration | | Slope cropland (Sc) | Natural restoration | |
| --- | --- | --- | --- | --- | --- |
|  | Artificial forest (Af) | Artificial grassland (Ag) |  | Natural grassland (Ng) | Natural shrub  (Ns) |
| Soil properties | - | - | - | - | - |
| pH | 7.07±0.06^a^ | 6.89±0.09^b^ | 7.11±0.05^a^ | 6.27±0.02^c^ | 6.08±0.03^c^ |
| SOC | 15.74±2.61^b^ | 10.26±1.92^c^ | 9.01±1.02^c^ | 14.58±0.85^b^ | 19.01±2.14^a^ |
| TN | 1.04±0.23^c^ | 0.75±0.15^d^ | 0.61±0.08^d^ | 1.56±0.14^b^ | 1.74±0.25^a^ |
| TP | 0.76±0.03^a^ | 0.51±0.02^a^ | 0.54±0.02^a^ | 0.98±0.09^a^ | 1.03±0.14^a^ |
| AP | 1.76±0.12^a^ | 1.78±0.23^a^ | 1.75±0.14^a^ | 1.87±0.25^a^ | 1.91±0.23^a^ |
| NH_4_^+^-N | 27.84±2.56^b^ | 20.79±3.12^c^ | 21.57±2.01^c^ | 26.84±1.89^b^ | 35.67±3.02^a^ |
| MBC | 389.11±23.62^b^ | 256.34±35.85^d^ | 198.77±12.85^e^ | 320.51±23.52^c^ | 515.26±36.53^a^ |
| MBN | 31.05±2.03^c^ | 30.49±2.17^c^ | 23.69±1.58^d^ | 36.98±3.6^2b^ | 56.03±2.11^a^ |
| Plant properties | - | - | - | - | - |
| Litter | 87.24±8.65^b^ | 26.78±3.54^c^ | 23.56±2.79^c^ | 95.23±9.12^b^ | 123.45±12.78^a^ |
| Coverage | 95.5±5.6^a^ | 68.3±7.9^b^ | 42.8±3.5^d^ | 59.9±6.8^c^ | 67.2±8.4^b^ |
| AGB | 1722.1±123.5^a^ | 41.5±3.8^d^ | 49.8±6.9^d^ | 166.4±12.6^c^ | 1122.9±156.6^b^ |
| *H*_plant_ | 1.54±0.23^c^ | 1.05±0.12^d^ | 2.35±0.16^b^ | 2.89±0.35^a^ | 2.76±0.24^a^ |
| *S*_plant_ | 5.12±0.52^c^ | 6.58±0.69^c^ | 9.78±0.85^b^ | 12.35±1.23^a^ | 13.18±1.07^a^ |

Note: pH: soil pH value; SOC: soil organic carbon; TN: soil total nitrogen; TP: soil total phosphorus; AP: soil available phosphorus; NH_4_^+^-N: soil NH_4_-N; MBC: soil microbial biomass C; MBN: soil microbial biomass N; AGB: aboveground biomass; *H*_plant_: Shannon-Wiener index; *S*_plant_: Patrick index

**Suppl. Table 3** Illumina MiSeq sequenced fungal data and fungal diversity diversity indices (at 97% sequence similarity) based on the ITS rRNA gene.

| Item | Artificial restoration | | Slope cropland  (Sc) | Natural restoration | |
| --- | --- | --- | --- | --- | --- |
|  | Artificial forest  (Af) | Artificial grassland  (Ag) |  | Natural grassland  (Ng) | Natural shrub  (Ns) |
| Quality sequences | 30152±1569 | 29872±2017 | 29254±2561 | 30879±3269 | 32789±1587 |
| Fungal sequences | 29897±1258 | 18974±1623 | 28568±1598 | 29987±2104 | 31520±2098 |
| Number of phylotype | 737±25^b^ | 659±23^c^ | 557±18^c^ | 835±12b | 927±20^a^ |
| Shannon's diversity | 6.98±0.13^a^ | 6.21±0.17^b^ | 5.56±0.52^c^ | 6.53±0.23^ab^ | 7.33±0.42^a^ |
| Simpson's diversity | 0.98±0.01^a^ | 0.95±0.01^a^ | 0.92±0.03^a^ | 0.97±0.02^a^ | 0.99±0.02^a^ |
| ACE index | 714±23^b^ | 623±25^c^ | 594±19^d^ | 702±13^b^ | 869±26^a^ |
| Good's coverage (%) | 99.41±5.23^a^ | 99.23±6.01^a^ | 99.52±3.04^a^ | 99.62±1.02^a^ | 99.61±2.15^a^ |
| Chao 1 index | 429±23^a^ | 431±12^a^ | 385±15^b^ | 432±18^a^ | 437±22^a^ |

Note: The data were calculated from 19000 fungal sequences per soil sample. Different letters within the same column indicate significant difference between treatments in individual sampling time tested by one-Way ANOVA (*p* < 0.05). The same below

**Suppl. Table 4** Relative abundances (%) of the dominant fungal genera (more than 1%) among different land use types.

| Item | Artificial restoration | | Slope cropland  (Sc) | Natural restoration | |
| --- | --- | --- | --- | --- | --- |
|  | Artificial forest  (Af) | Artificial grassland  (Ag) |  | Natural grassland  (Ng) | Natural shrub  (Ns) |
| *Mortierella* | 16.16±1.36^c^ | 20.43±2.05^a^ | 18.11±1.02^b^ | 15.16±2.04^c^ | 16.38±1.87^c^ |
| *Fusarium* | 10.01±1.03^b^ | 13.10±1.58^a^ | 7.54±0.56^c^ | 6.05±1.17^c^ | 14.74±1.36^a^ |
| *Guehomyces* | 11.09±1.26^d^ | 10.85±1.25^d^ | 13.08±2.31^c^ | 22.44±2.87^a^ | 18.32±1.04^b^ |
| *Cryptococcus* | 7.30±0.86^a^ | 6.77±0.65^a^ | 7.99±1.26^a^ | 5.98±0.98^a^ | 6.45±0.69^a^ |
| *Corynespora* | 7.79±1.23^a^ | 2.28±0.56^c^ | 5.13±0.85^b^ | 3.57±0.65^c^ | 2.53±0.05^c^ |
| *Schizothecium* | 6.27±0.87^c^ | 7.54±0.24^b^ | 9.77±1.54^a^ | 7.83±0.45^ab^ | 8.83±0.78^ab^ |
| *Ustilago* | 2.10±0.23^a^ | 0.05±0.01^b^ | 0.03±0.00^b^ | 0.04±0.01^b^ | 0.05±0.02^b^ |
| *Phoma* | 1.97±0.56^b^ | 4.59±0.57^a^ | 3.84±0.36^ab^ | 3.70±0.58^b^ | 2.01±0.56^b^ |
| *Nectria* | 1.78±0.45^b^ | 5.52±0.68^a^ | 1.02±0.02^c^ | 0.91±0.12^c^ | 1.89±0.65^b^ |
| *Tetracladium* | 1.38±0.29^b^ | 1.42±0.23^b^ | 1.06±0.04^b^ | 2.27±0.25^a^ | 1.20±0.52^b^ |
| *Gliomastix* | 1.34±0.35^a^ | 0.62±0.07^b^ | 0.38±0.05^b^ | 0.53±0.04^bc^ | 0.87±0.14^b^ |
| *Microdochium* | 1.29±0.57^a^ | 0.92±0.09^ab^ | 1.16±0.06^ab^ | 0.39±0.06^b^ | 0.70±0.05^ab^ |
| *Ceratobasidium* | 1.20±0.32^b^ | 0.59±0.03^c^ | 0.24±0.03^c^ | 2.38±0.14^a^ | 1.03±0.09^b^ |
| *Geminibasidium* | 1.15±0.12^ab^ | 1.09±0.05^ab^ | 1.41±0.21^a^ | 0.90±0.08^b^ | 0.71±0.02^b^ |
| *Thelebolus* | 0.91±0.09^a^ | 0.53±0.06^b^ | 0.54±0.11^b^ | 0.56±0.06^b^ | 0.90±0.06^a^ |
| *Penicillium* | 0.87±0.07^a^ | 0.23±0.02^b^ | 0.41±0.09^b^ | 0.59±0.07^b^ | 0.35±0.04^b^ |

**Suppl. Table 5** Spearman's correlations (*r*) between soil fungal diversity, plant and soil properties determined by Mantel test. *p* values were calculated based on 999 permutations. Total: the Spearman's correlation among plant and soil properties and fungal diversity structure, irrespective of land use type.

| Factors | Variance explained  (70%) | Artificial restoration | | | | Slope cropland  (Sc) | | Natural restoration | | | |
| --- | --- | --- | --- | --- | --- | --- | --- | --- | --- | --- | --- |
|  |  | Artificial forest  (Af) | | Artificial grassland  (Ag) | |  |  | Natural grassland  (Ng) | | Natural shrub  (Ns) | |
|  |  | *r* | *p* | *r* | *p* | *r* | *p* | *r* | *p* | *r* | *p* |
| Soil properties |  | - | - | - | - | - | - | - | - | - | - |
| pH | 15.23% | -0.785 | <0.001 | -0.623 | <0.001 | -0.518 | <0.05 | -0.659 | <0.001 | -0.894 | <0.001 |
| SOC | 14.02% | 0.789 | <0.001 | 0.787 | <0.001 | 0.799 | <0.001 | 0.814 | <0.001 | 0.889 | <0.001 |
| TN | 5.62% | 0.402 | >0.05 | 0.402 | >0.05 | 0.325 | >0.05 | 0.488 | <0.05 | 0.412 | >0.05 |
| TP | 0.26% | -0.326 | >0.05 | 0.058 | >0.05 | 0.127 | >0.05 | -0.169 | >0.05 | 0.263 | >0.05 |
| AP | 0.98% | 0.069 | >0.05 | -0.145 | >0.05 | 0.087 | >0.05 | 0.158 | >0.05 | -0.257 | >0.05 |
| NH_4_^+^-N | 7.89% | 0.567 | <0.05 | 0.553 | <0.05 | 0.628 | <0.001 | 0.577 | <0.05 | 0.612 | <0.01 |
| MBC | 12.71% | 0.699 | <0.001 | 0.712 | >0.05 | 0.703 | <0.001 | 0.814 | <0.001 | 0.823 | <0.001 |
| MBN | 10.43% | 0.723 | <0.001 | 0.689 | <0.001 | 0.715 | <0.001 | 0.756 | <0.001 | 0.817 | <0.001 |
| Plant properties | - | - | - | - | - | - | - | - | - | - | - |
| Litter | 16.87 | 0.799 | <0.001 | 0.742 | <0.001 | 0.621 | <0.01 | 0.894 | <0.001 | 0.823 | <0.001 |
| Coverage | 10.25 | 0.685 | <0.001 | 0.798 | <0.001 | 0.623 | <0.01 | 0.801 | <0.001 | 0.756 | <0.001 |
| AGB | 15.32 | 0.657 | <0.001 | 0.523 | <0.05 | 0.514 | <0.05 | 0.621 | <0.01 | 0.793 | <0.001 |
| *H*_plant_ | 0.75 | 0.236 | >0.05 | 0.326 | >0.05 | 0.205 | >0.05 | 0.207 | >0.05 | 0.541 | <0.05 |
| *S*_plant_ | 3.04 | 0.147 | >0.05 | 0.201 | >0.05 | 0.395 | >0.05 | 0.146 | >0.05 | 0.089 | >0.05 |

**Suppl. Table 6** Direct, indirect and total effects on fungal diversity based on the standardized values of statistically significant SEM paths (*F=*35.26, df=30, *p*<0.01, GFI=0.087, AIC=116.35, RSMEA=0.024, 64.32%)

| Item | | Direct pathway effect | Indirect pathway effect | Total effect |
| --- | --- | --- | --- | --- |
| Soil properties | pH | 0.123 | 0.025 | 0.148 |
|  | SOC | 0.236 | 0.047 | 0.283 |
|  | TN | 0.124 | 0.000 | 0.124 |
|  | TP | 0.000 | 0.059 | 0.059 |
|  | AP | 0.012 | 0.041 | 0.053 |
|  | NH_4_^+^-N | 0.156 | 0.000 | 0.156 |
|  | MBC | 0.000 | 0.024 | 0.024 |
|  | MBN | 0.000 | 0.033 | 0.033 |
| Plant properties | Litter | 0.000 | 0.156 | 0.156 |
|  | Coverage | 0.000 | 0.087 | 0.087 |
|  | AGB | 0.159 | 0.000 | 0.159 |
|  | *H*_plant_ | 0.056 | 0.224 | 0.280 |
|  | *S*_plant_ | 0.023 | 0.072 | 0.095 |
| Fungal  diversity | Shannon's diversity | 0.163 | 0.149 | 0.312 |
|  | Simpson's diversity | 0.058 | 0.052 | 0.110 |
|  | ACE index | 0.123 | 0.078 | 0.201 |
|  | Coverage (%) | 0.078 | 0.000 | 0.078 |
|  | Chao 1 index | 0.164 | 0.021 | 0.185 |
